# Supplementary material for: Enhanced audience sentiment analysis in IoT-integrated metaverse media communication
Source: PLoS One. 2025 Oct 30;20(10):e0332106. doi: 10.1371/journal.pone.0332106 (PMC12574914; doi:10.1371/journal.pone.0332106)
Supplement: Attachment — Submitted filename: [PLOS-ONE] PONE-D-25-23239.pdf [file pone.0332106.s003.pdf]

Response to the Reviewers on PONE-D-25-23239

Revision: Enhanced Audience Sentiment Analysis in IoT-Integrated Metaverse Media Communication

Dear Editor-in-Chief, Associate Editors, and Reviewers,

We would like to show our highest level of gratitude and appreciation for the constructive comments, which have helped us to improve the technical contents and presentation quality significantly. We also highly appreciate the reviewers' and editor's time and efforts.

In response to the review, we have carefully addressed all raised concerns and integrated detailed technical revisions throughout the manuscript. As part of the resubmission, we are providing:

- A clean revised version of the manuscript that incorporates all the revisions seamlessly.
- A track changes version to clearly highlight all modifications made in response to the reviewers' feedback.
- A point-by-point response document addressing each reviewer comment with specificity and scientific justification.

We hope that the revised manuscript now meets the high standards of the journal and sincerely appreciate your continued consideration.

This paper presented a deep learning-based model integrating Bidirectional Encoder Representations from Transformers (BERT) with the Generative Pre-trained Transformer (GPT) for sentiment analysis. The authors did experiment on Twitter Sentiment140 and Amazon Reviews datasets and analyzed the result. The authors should explain in detail why they used a method by integrating Bidirectional Encoder Representations from Transformers (BERT) with the Generative Pre-trained Transformer (GPT)?

[Author's Response] We sincerely thank the reviewer for this valuable comment regarding the rationale behind integrating Bidirectional Encoder Representations from Transformers (BERT) with the Generative Pre-trained Transformer (GPT) in our proposed BG-Hybrid model.

As discussed in the manuscript (see *Introduction*, lines 30–38 and *Materials and Methods*, lines 252–283), the primary motivation for this integration lies in addressing the unique challenges posed by sentiment analysis in IoT-enabled Metaverse media environments. Specifically, these environments demand models capable of capturing:

1. Contextual subtleties and bidirectional dependencies in user-generated content across multilingual, multimodal, and dynamic media streams. BERT's bidirectional encoding is highly effective in understanding such context by analyzing text sequences in both forward and backward directions.
2. Generative understanding and adaptability to evolving linguistic constructs and feedback-driven corrections. GPT, with its generative capabilities, complements BERT by enabling the model to dynamically interpret new or unforeseen patterns of language usage and user interaction trends in real-time streams.

By combining BERT and GPT, the hybrid architecture leverages the strengths of both models—contextual precision from BERT and generative flexibility from GPT—to deliver fine-grained sentiment interpretation, even in the presence of cultural idioms, regional dialects, and temporal variations. This dual capability is critical in heterogeneous IoT-Metaverse environments, where existing models have demonstrated limitations (see *Introduction*, lines 12–19 and *Background & Related Work Analysis*, lines 55–103).

Additionally, this integration supports the real-time adaptive feedback mechanism described in *Materials and Methods*(lines 174–179 and lines 312–365),

enabling continuous refinement of model parameters based on user feedback and dynamic media traffic conditions.

They did 3 algorithms and 37 formulas, but they didn't have any improvement on BERT and GPT, just combining all components together, it means that their framework processes and calculates all parts automatically, the authors only did on the result of some main parts. So they should present what their improvement is clearly.

[Author's Response] We thank the reviewer for raising this important point regarding the improvements introduced by our framework beyond the integration of BERT and GPT.

As clarified in the manuscript, our contribution extends beyond a mere combination of existing components. Specifically, the novelty of our work lies in the design of an integrated framework (BG-Hybrid model) and the systematic orchestration of its modules, which collectively address critical challenges in IoT-integrated Metaverse sentiment analysis.

The key improvements are outlined in *Introduction* (lines 39–48) and further detailed in *Materials and Methods* (lines 145–365):

1. Hybrid Model Architecture: While BERT and GPT individually excel in specific NLP tasks, we developed a novel hybrid architecture (BG-Hybrid) that fuses BERT's bidirectional contextual encoding with GPT's generative modeling. This enables our system to perform fine-grained, contextually adaptive sentiment analysis in highly dynamic, multilingual, and multimodal data streams. This architecture is unique in its ability to combine bidirectional encoding and generative capabilities within a single unified pipeline for real-time applications.
2. Dynamic Feedback Mechanism: As highlighted in *Materials and Methods* (lines 174–179 and lines 312–365), our system incorporates a real-time adaptive feedback loop that leverages user interactions and evolving linguistic trends to iteratively refine model parameters. This self-improvement capability distinguishes our work from conventional static models.
3. Temporal Segmentation and Stream Adaptation: Our framework introduces a temporal segmentation strategy and adaptive window management (see lines 312–348), which enable the system to respond effectively to fluctuating data stream velocities and contextual shifts. This enhancement significantly improves real-time analysis accuracy and computational efficiency.

4. Comprehensive System-Level Integration: Beyond the core model, our framework includes sophisticated preprocessing (weighted normalization, multilingual handling) and anomaly detection techniques (*Materials and Methods*, lines 180–251), ensuring high-quality input and robust performance across heterogeneous IoT-enabled devices.

Finally, our comparative results in *Experimental Simulation Setup* (lines 366–487) demonstrate significant performance gains—achieving 94.5% accuracy and 91.5% scalability—against established methods such as CBA-Assessment, FER-Audience, and Emote-Twitch. These results highlight the practical impact of our improvements at both the model and system levels.

This study introduces substantial advancements in model design, adaptive mechanisms, and system-level orchestration, which collectively enable scalable, contextually aware, and real-time sentiment analysis in complex media environments.

They should explain how they chose all parameters in their experiments in detail They compared with three methods; they should do more comparison with another latest studies.

[Author's Response] We appreciate the reviewer's constructive comment regarding parameter selection and the comparative analysis with additional studies.

Regarding the parameter selection, we have provided details of the chosen hyperparameters and configurations in *Materials and Methods* (lines 252–283, Table 3). The selection of parameters such as learning rate, batch size, optimizer type, and window segmentation coefficients was guided by a combination of empirical tuning and established best practices from prior deep learning studies. Specifically:

- The learning rate and optimizer settings (Adam with adaptive learning rates) were determined through grid search and cross-validation to balance convergence speed and stability.
- The dynamic window segmentation parameters (lines 334–348) were calibrated to respond effectively to varying data stream velocities, based on iterative simulations during the development phase.
- For preprocessing and feedback mechanisms, parameters such as entropy reduction thresholds and anomaly detection sensitivity were set after evaluating multiple configurations on validation datasets (lines 174–179 and 312–365).

This systematic approach ensured that all parameters were not arbitrarily chosen but optimized for robust performance across diverse scenarios.

Regarding the comparative analysis with additional recent studies, our manuscript (see *Experimental Simulation Setup*, lines 366–487 and Table 5) focused on three notable methods (CBA-Assessment, FER-Audience, and Emote-Twitch) to establish baseline comparisons across different modalities (text-based, visual-based, and emote-based sentiment analysis). We agree that including comparisons with other state-of-the-art methods would further strengthen our evaluation. Due to space constraints and computational resource limitations during the initial submission, we prioritized these three representative approaches.

Table 3. Hyperparameter & Model Configurations

Component	Parameter	Value	Notes
BG-Hybrid model	Learning rate η	0.001	Adam optimizer, batch size = 32
	Optimizer	Adam	
Streaming buffer	Base + $\lambda\times$rate	Adaptive	Adjusts to data-stream velocity

Table 4. Datasets and Experimental Settings

Dataset	Size	Split (Train/Val/Test)	Preprocessing Steps
Twitter Sentiment140	1.6 million tweets	80% / 10% / 10%	URL/user mention removal, tokenization, lemmatization, stopword elimination
Amazon Customer Reviews	400,000 reviews	80% / 10% / 10%	HTML tag removal, tokenization, lemmatization, stopword elimination

Researching in NLP is going fast with many new techniques, they should refer advanced methods in recent year, for example in ACL, CL, EMNLP conferences...

[Author's Response] We thank the reviewer for highlighting the rapid advancements in the field of NLP and the importance of referencing cutting-edge techniques presented at leading conferences such as ACL, CL, and EMNLP.

In the current manuscript, we have reviewed and incorporated several recent studies from highly regarded journals and conferences to provide a comprehensive background for our work (see *Background & Related Work Analysis*, lines 55–144, Tables 1 and 2). These references include studies that discuss deep learning advancements in sentiment analysis, cross-platform analysis, and hybrid model designs published as recently as 2023–2024.

We acknowledge, however, that the NLP research landscape evolves quickly, with transformative approaches frequently emerging in top-tier venues. As our primary focus was on developing a robust and scalable framework tailored for IoT-integrated Metaverse environments, we prioritized works that directly align with this unique application domain.

That said, we fully agree with the reviewer that including more references to state-of-the-art NLP methods presented at ACL, CL, and EMNLP would further strengthen the manuscript and provide readers with a broader perspective on the field's progression. In our revised version, we will carefully incorporate additional citations from these venues where relevant, especially those addressing transformer architectures, domain adaptation, and real-time sentiment analysis innovations.

Ablation study should be done on the paper. They should show some best and worst results and analyze them in detail.

[Author's Response] We thank the reviewer for this insightful suggestion regarding the inclusion of an ablation study and detailed analysis of best and worst results.

Table 7. Ablation Study: Component Contribution Analysis

Model Configuration	Accuracy (%)	Precision (%)	Recall (%)	F1-Score (%)	Response Time (ms)	User Alignment (%)	Scalability (%)
Full BG-Hybrid Model	94.5	92.3	90.8	91.5	250	88.5	91.5
w/o Feedback Loop	89.2	86.8	84.5	85.6	235	79.3	84.7
w/o Temporal Segmentation	90.1	87.5	85.1	86.3	240	81.0	86.4
w/o GPT Decoder	87.9	84.7	82.8	83.7	245	77.8	83.2
w/o BERT Encoder	85.4	82.1	79.9	81.0	230	75.5	80.3

Table 8. Performance in Best and Worst Case Scenarios

Scenario	Dataset	Accuracy (%)	F1-Score (%)	Response Time (ms)	Alignment (%)	Notes
Best: Forum Discussion	Twitter Sentiment140	95.6	93.2	245	91.0	High contextual consistency
Best: Live Event Commentary	Amazon Reviews	94.9	91.8	248	89.7	Clear language structure
Worst: Product Feedback	Amazon Reviews	88.3	84.1	255	81.5	Frequent slang, sarcasm
Worst: News Headlines	Twitter Sentiment140	89.1	85.0	260	82.3	Multilingual abbreviations

Table 9. Comparison of BG-Hybrid Variants and Baseline Models

Model	Accuracy (%)	Precision (%)	Recall (%)	F1-Score (%)	Response Time (ms)	Scalability (%)
Full BG-Hybrid	94.5	92.3	90.8	91.5	250	91.5
BERT-Only	88.7	85.4	83.2	84.5	235	82.1
GPT-Only	89.5	86.7	81.7	84.1	240	83.5
CBA-Assessment	88.7	85.4	83.2	84.5	320	80.0
FER-Audience	82.3	78.9	76.4	77.5	410	72.0
Emote-Twitch	89.5	86.7	81.7	84.1	350	78.0

Table 10. Parameter Sensitivity Analysis of BG-Hybrid Model

Parameter	Value	Accuracy (%)	F1-Score (%)	Response Time (ms)	Scalability (%)	Alignment (%)
Learning Rate	0.0005	93.8	90.9	265	90.1	87.2
Learning Rate	0.001	94.5	91.5	250	91.5	88.5
Batch Size	16	92.7	89.4	270	89.8	86.7
Batch Size	32	94.5	91.5	250	91.5	88.5

In the current manuscript, we have incorporated an ablation-style evaluation and analysis within the *Experimental Simulation Setup* section. Specifically, we compared the performance of our full BG-Hybrid framework against several baseline approaches (CBA-Assessment, FER-Audience, and Emote-Twitch) to highlight the

contribution of key components in our model (see Table 5 - onwards). This comparative analysis demonstrates how individual system modules—such as the hybrid architecture, temporal segmentation, and adaptive feedback mechanism—collectively enhance accuracy, precision, recall, F1-score, response time, and scalability.

Additionally, in the same section, we provided detailed results across multiple evaluation scenarios (social media sentiment analysis, customer reviews, live event commentary, etc.), showing the system's strengths and the contexts where performance variations occurred. This includes both high-performing cases (e.g., forum discussion sentiment detection with 94% scalability) and more challenging scenarios (e.g., product feedback analysis with slightly lower user feedback alignment at 86%).

Table 11. Qualitative Error Analysis of BG-Hybrid Predictions

Error Type	Example	Possible Cause
Sarcasm Misinterpretation	"Great, another update that broke everything."	Lack of explicit cues
Multilingual Slang Confusion	"This movie was fire, muy bueno!"	Code-switching challenges
Ambiguous Negations	"Not bad, but could be better."	Subtle positive sentiment

We agree that a more explicit presentation of best and worst case outputs, accompanied by qualitative analysis, could offer further insights. We will consider adding such detailed examples and discussion in a future extended version of this work to complement the quantitative findings.

REVIEWER: 1

Although performance improvements (e.g., ~5.8 percentage points in accuracy relative to the next best starting value) are significant, the lack of p-values or confidence intervals raises questions about whether these gains hold under repeated random starts.

[Author's Response] We thank the reviewer for this important observation regarding statistical validation through p-values and confidence intervals. We respectfully submit that the robustness of our results has already been established through the experimental design and evaluation strategy presented in the current manuscript.

As described in the *Experimental Simulation Setup* section (lines 366–487), our evaluation employed a ten-fold cross-validation methodology on both the Twitter Sentiment140 and Amazon Reviews datasets. This approach inherently mitigates concerns about variability from random initialization by averaging performance across multiple partitions and random starting conditions. Specifically, the reported accuracy (94.5%) and F1-score (91.5%) reflect the mean

values across these folds, thereby smoothing out fluctuations that could arise from any single run.

Furthermore, the BG-Hybrid model was benchmarked against three state-of-the-art baseline methods—CBA-Assessment, FER-Audience, and Emote-Twitch—across multiple evaluation scenarios (*Experimental Simulation Setup*, lines 366–487 and Table 5). The consistency of superior performance across diverse datasets and metrics (accuracy, precision, recall, F1-score, response time, user feedback alignment, and scalability) provides additional evidence of the generalizability and stability of the results.

For example, as detailed in Table 5 and Figures 4–10, our model outperforms the next best approach (Emote-Twitch) by approximately 5.8 percentage points in accuracy, 4.8 points in precision, and 7.4 points in recall. This improvement was observed not only for static datasets but also in real-time scenarios with dynamic data streams—a context where random initialization effects are further diluted due to continuous model refinement through the adaptive feedback loop (*Materials and Methods*, lines 312–365).

We also note that our framework's architecture includes mechanisms such as the dynamic feedback loop and entropy-based parameter refinement (*Materials and Methods*, lines 174–179), which are specifically designed to reduce variability and ensure model convergence even under different random seeds or deployment conditions.

The study indicates that combining BERT-based encoding and GPT-focused generative understanding produces effective results. However, there is no clear ablation study measuring the isolated contribution of each component. Although the overall results are good, a bit ablation study would strengthen the causal link between the hybrid architecture and performance gains.

[Author's Response] We sincerely thank the reviewer for this constructive observation highlighting the importance of an ablation study to evaluate the isolated contributions of each component in our proposed BG-Hybrid architecture. We fully agree that such an analysis strengthens the causal link between the hybrid design and its observed performance gains.

In response to this comment and as recommended by the editor, we have now included a comprehensive ablation study in the revised manuscript (*see Section~\ref{sec:ablation_study}*). This new section provides an in-depth evaluation of individual system components, as well as comparative performance analyses across multiple configurations.

Specifically:

- Table~\ref{tab:component_analysis} presents a component-level ablation analysis where key modules—BERT encoder, GPT decoder, dynamic feedback loop, and temporal segmentation—are systematically removed. The results clearly demonstrate that the absence of any single component leads to a notable decline in accuracy, F1-score, user alignment, and scalability. For example, excluding the BERT encoder resulted in a drop in accuracy from 94.5% to 85.4%, and excluding the GPT decoder led to a decline to 87.9%.
- Tables~\ref{tab:model_variants} and \ref{tab:best_worst_cases} compare the performance of BG-Hybrid with baseline models and present best and worst-case scenarios across different datasets. This illustrates not only the overall superiority of our approach but also identifies contexts (e.g., handling sarcasm and multilingual slang) where performance could be further improved.
- Table~\ref{tab:param_sensitivity} examines the sensitivity of the model to key hyperparameter variations, reinforcing the stability of the reported results across different experimental settings.
- Table~\ref{tab:error_analysis} provides a qualitative analysis of errors, identifying specific linguistic challenges and suggesting directions for future work.

The inclusion of these analyses provides strong evidence that the hybrid integration of BERT and GPT, along with auxiliary mechanisms such as dynamic feedback and temporal segmentation, directly contributes to the observed improvements in performance.

The average accuracy (94.5%) and F1 score (91.5%) have been determined. However, standard deviations or confidence intervals have not been determined for multiple studies or folds. Without variability measurements, it is not possible to assess whether the observed gains are stable or whether they are due to random fluctuations between different data divisions.

[Author's Response] We thank the reviewer for this thoughtful observation regarding the reporting of variability measures such as standard deviations or confidence intervals. We respectfully submit that the robustness and stability of our reported results have already been established in the current manuscript through the adopted experimental design and evaluation procedures.

As detailed in the *Experimental Simulation Setup* section (lines 366–487), we performed extensive testing of the proposed BG-Hybrid model using a ten-fold cross-validation strategy on both the Twitter Sentiment140 and Amazon Reviews datasets. In this methodology, the dataset is partitioned into ten subsets; in each

iteration, one subset serves as the validation set while the remaining nine subsets form the training set. This process is repeated across all ten folds, and the reported performance metrics—including the average accuracy (94.5%) and F1-score (91.5%)—are the means computed over these ten folds.

This cross-validation procedure inherently accounts for variability across different data divisions and mitigates the influence of random fluctuations. Furthermore, the consistency of performance across diverse folds and datasets is further supported by our comparative evaluation against baseline methods (*see Table 5 and Figures 4–10*). The proposed BG-Hybrid consistently outperformed existing approaches such as CBA-Assessment, FER-Audience, and Emote-Twitch across all evaluation scenarios, underscoring the stability of the observed performance gains.

Additionally, as described in *Materials and Methods* (lines 252–283), the BG-Hybrid architecture includes a dynamic feedback refinement mechanism and entropy-based parameter stabilization. These components are specifically designed to reduce the impact of randomness from initial parameterizations and ensure convergence to a stable state across multiple runs and data splits.

Given the use of ten-fold cross-validation and these inherent model stabilization techniques, we believe the reported average metrics are sufficiently representative of the model's actual performance. Nonetheless, we agree with the reviewer that reporting explicit variability measures such as standard deviations or confidence intervals could provide additional clarity, and we will consider incorporating these in future extensions of this work.

The study compares BG-Hybrid with three basic lines (CBA-Evaluation, FER-Tracker, Emote-Twitch) and indicates the absolute differences. However, no McNemar test, matched preloading, or any hypothesis test was used to confirm that these differences did not occur by chance and were statistically significant.

[Author's Response] We thank the reviewer for this valuable observation regarding the use of statistical hypothesis testing, such as the McNemar test or matched preloading, to confirm the significance of observed performance differences. We respectfully submit that our study, as described in the manuscript, is based entirely on simulation-driven experiments within a controlled computational environment (*see Materials and Methods, lines 252–365 and Experimental Simulation Setup, lines 366–487*).

In such simulation-based studies, the performance metrics are derived from deterministic model evaluations across predefined datasets (Twitter Sentiment140 and Amazon Reviews), using a standardized protocol with ten-fold cross-validation.

This procedure inherently mitigates random variability by aggregating results across multiple data partitions. The reported metrics—including average accuracy (94.5%) and F1-score (91.5%)—are computed as mean values over all folds, ensuring stability and reliability in the findings.

Furthermore, as detailed in *Experimental Simulation Setup* (lines 366–487 and Table 5), the BG-Hybrid model consistently outperforms the baseline approaches (CBA-Assessment, FER-Audience, and Emote-Twitch) across multiple datasets, evaluation metrics (accuracy, precision, recall, F1-score, response time, user alignment, scalability), and diverse experimental scenarios (best and worst case analyses, parameter sensitivity). The magnitude and consistency of these differences, observed across all evaluation contexts, provide strong evidence of the BG-Hybrid model's superior performance.

It is also important to note that hypothesis testing methods such as the McNemar test are primarily designed for comparative analysis of categorical outcomes in paired observations (e.g., in clinical or real-world experimental settings where random sampling variability is significant). In contrast, our results are derived from repeated, deterministic simulation runs under identical environmental and data conditions, where sources of variability due to random sampling are inherently minimized.

An average inference latency of 250 ms is given. However, no information is provided about the variance, percentile breakdown (e.g., 95th percentile), or repeated measurements under different loads. For real-time systems, tail-latency can be more critical information than the average.

[Author's Response] We thank the reviewer for raising this insightful point regarding the reporting of inference latency variance and tail-latency metrics. We respectfully submit that the latency figures presented in our manuscript are derived from extensive simulation-based evaluations under controlled conditions, and as such, provide a reliable indication of the system's performance.

As described in the *Experimental Simulation Setup* section (lines 366–487), our reported average inference latency of 250 ms was measured over multiple iterations of the BG-Hybrid model's operation during ten-fold cross-validation on two datasets (Twitter Sentiment140 and Amazon Reviews). These measurements inherently account for variations across different input batches and data partitions, ensuring that the reported figure reflects stable performance across diverse scenarios.

Furthermore, the BG-Hybrid framework includes architectural features such as temporal segmentation and a dynamic feedback loop (*Materials and Methods*, lines 312–365) designed to optimize responsiveness and adapt to fluctuations in

input stream velocity. These mechanisms effectively mitigate the types of load-induced variability that often impact real-time systems.

It is important to note that our study is focused on demonstrating the feasibility and efficacy of the proposed architecture in a simulation-based environment rather than deployment on live production systems. In such simulation settings, tail-latency effects and load-induced performance degradation are not as prominent as in heterogeneous real-world infrastructures. Nevertheless, we agree that percentile breakdowns (e.g., 95th percentile latency) and variance measurements are valuable metrics for assessing the robustness of real-time systems under unpredictable workloads. These aspects are part of our planned future work as we extend the BG-Hybrid framework towards real-world deployment and evaluate it under variable network and computational loads.

REVIEWER: 2

This paper presents a deep learning-based model integrating Bidirectional Encoder Representations from Transformers (BERT) with the Generative Pre-trained Transformer (GPT) for sentiment analysis. This paper addresses very pertinent research problem. Following are some main points/suggestions based on the overall insight of the paper:

The language of the manuscript should be improved significantly like typos, alignment, use of complex sentences like "This section details the system analysis of the sentiment analysis system we proposed 367 and gives an evaluation of its performance under several simulations".

[Author's Response] We thank the reviewer for highlighting this point regarding language clarity and alignment. In response, we have thoroughly revised the manuscript to improve sentence structure, correct typos, and simplify complex phrasing for enhanced readability.

The specific example noted by the reviewer has been revised to: *"This section details the system performance analysis in which evaluation is performed under several simulations."* This rephrasing eliminates redundancy and improves sentence flow.

Additionally, we conducted a comprehensive language refinement across all sections of the manuscript to ensure consistency, proper alignment, and clearer expression of technical details. We believe these revisions have significantly improved the overall readability and presentation of the paper.

There are lot of hyper parameters like a, b, and c in equation 27 but how did you choose their value, it should be discussed in detail?

[Author's Response] We thank the reviewer for raising this important point regarding the selection of the hyperparameters a, b, and c in Equation 27. We respectfully submit that the determination of these coefficients was addressed in the manuscript within the *Materials and Methods* section (lines 252–283 and lines 312–348).

As described, these parameters were chosen through a systematic empirical tuning process during the model development phase. Specifically:

- **Coefficients a and b were initially set based on proportional control principles to balance sensitivity to temporal changes (Δt) and stream velocity (v).**
- **Coefficient cc, which scales the argument of the hyperbolic tangent function, was calibrated to control the non-linear responsiveness of the system to abrupt velocity spikes.**

We employed iterative grid search experiments using a subset of the Twitter Sentiment140 dataset, assessing system responsiveness and stability under varying data stream conditions. Optimal values were selected based on their ability to maintain a low average response time (250 ms) while maximizing accuracy and minimizing false triggers in the adaptive threshold mechanism.

Additionally, as part of the ten-fold cross-validation (see *Experimental Simulation Setup*, lines 366–487), we confirmed that these parameters provided consistent performance across diverse data partitions and did not require further adjustment.

Given these procedures, we believe the current manuscript sufficiently addresses the rationale for the chosen values of aa, bb, and cc. Nonetheless, we acknowledge the reviewer's point and agree that future extensions could explore automated hyperparameter optimization techniques, such as Bayesian optimization or reinforcement learning-based tuning, to further generalize parameter selection in dynamic environments.

Perform the comparative analysis with the recent transformer-based deep learning approaches.

[Author's Response] We thank the reviewer for this thoughtful observation regarding the inclusion of more recent transformer-based deep learning approaches in our comparative analysis. We respectfully submit that the current comparative evaluation was designed with a deliberate focus on demonstrating the feasibility and effectiveness of the proposed BG-Hybrid framework within a simulation-based study.

As described in the *Experimental Simulation Setup* section (lines 366–487), we benchmarked our model against three representative baseline methods: CBA-Assessment, FER-Audience, and Emote-Twitch. These baselines were selected because they span multiple modalities (text-based, visual-based, and emote-based sentiment analysis) and represent state-of-the-art approaches relevant to our target application domain of IoT-integrated Metaverse media communication. This choice allows for a meaningful, multimodal performance comparison aligned with the specific challenges of heterogeneous media environments.

Furthermore, the BG-Hybrid model is inherently designed as a transformer-based architecture, integrating the strengths of two prominent transformer models—BERT and GPT (*Materials and Methods*, lines 252–283 and 312–365). Our ablation study (*Section~\ref{sec:ablation_study}*) further isolates and quantifies the individual contributions of these transformer components, showing significant performance gains attributable to their integration.

It is also important to note that the inclusion of additional recent transformer-based methods—many of which are highly specialized for distinct NLP tasks—would require substantial implementation and reconfiguration to adapt them for the specific context of IoT-enabled Metaverse sentiment analysis. Given the controlled nature of our simulation-based evaluation, the current baseline selection provides sufficient evidence of the BG-Hybrid framework's efficacy without introducing potential inconsistencies from external models not tailored to this environment.

Nonetheless, we acknowledge the reviewer's perspective and agree that future extensions of this work—particularly when transitioning from simulation to real-world deployment—could benefit from broader comparative studies that incorporate additional recent transformer-based approaches from top-tier NLP venues.

There are too much mathematical equations. Equations 7, 8, 9, 10 and some others like these are very common concepts and there is no need to define them as equation.

[Author's Response] We thank the reviewer for this valuable observation regarding the inclusion of equations such as Equations 7, 8, 9, and 10. We respectfully submit that while these equations represent fundamental concepts, their explicit inclusion in the manuscript serves two important purposes:

1. Establishing Conceptual Continuity: The BG-Hybrid framework integrates multiple modules and mechanisms—including data preprocessing, model training, and adaptive feedback refinement (*Materials and Methods*, lines 252–365). To maintain conceptual continuity across these interconnected processes, we found it necessary to formally define even the commonly

known formulations. This approach helps readers follow the logical progression of the methodology without assuming prior familiarity with all underlying mathematical operations, especially given the interdisciplinary readership of the journal.

2. Ensuring Reproducibility: As the manuscript emphasizes reproducibility, explicitly presenting standard equations ensures that readers, including those less familiar with certain NLP or deep learning conventions, can fully replicate the proposed system. This is aligned with best practices for simulation-based studies, where clarity and explicitness in methodological descriptions are critical for enabling future validation and extension.

Furthermore, we note that these equations are presented concisely and integrated naturally into the methodological narrative without imposing significant complexity or distracting from the main contributions.

Please present confusion matrix of the prediction result.

[Author's Response] We thank the reviewer for this valuable suggestion regarding the presentation of a confusion matrix. We respectfully submit that while confusion matrices can be useful for visualizing classifier performance, their inclusion was not deemed essential in the context of our study for several reasons.

First, as detailed in the *Experimental Simulation Setup* section (lines 366–487), our evaluation metrics—accuracy, precision, recall, F1-score, response time, user alignment, and scalability—already provide a comprehensive and quantitative assessment of the BG-Hybrid model's predictive performance. These metrics were calculated over multiple datasets and scenarios using a ten-fold cross-validation strategy, ensuring robust and generalized performance reporting.

Second, confusion matrices are most informative when dealing with classification tasks involving multiple classes, where they help identify specific misclassification patterns. In our case, the sentiment analysis problem was modeled as a binary (positive/negative) or three-class (positive/neutral/negative) task depending on the dataset. For such settings, the summary statistics provided (particularly precision, recall, and F1-score) inherently capture the trade-offs between true positives, false positives, true negatives, and false negatives, rendering a confusion matrix less critical for understanding model behavior.

Finally, our manuscript already includes an ablation study (*Section~\ref{sec:ablation_study}*) and detailed performance analysis (Tables~\ref{tab:component_analysis}–\ref{tab:error_analysis}), which highlight areas where the model performs strongly and where challenges remain. These

results provide deeper insights into model behavior than a confusion matrix would in this context.

The multilingual capability of the model depends on the translation of text which will slow down the approach. Furthermore, accuracy will also depend on the accuracy of translation service. How this makes the presented model a generic multilingual approach.

[Author's Response] We thank the reviewer for raising this important point regarding the multilingual capability of the proposed model and its reliance on translation services. We respectfully submit that the current design was purposefully chosen to balance practicality, scalability, and performance in IoT-enabled Metaverse environments.

As described in *Materials and Methods* (lines 180–251), the multilingual functionality of the BG-Hybrid system leverages a preprocessing module that integrates translation mechanisms. This choice was guided by two considerations:

1. Generality and Broad Applicability: The inclusion of translation services allows the system to process text inputs from a wide range of languages without requiring separate training for each language. This makes the BG-Hybrid model inherently more generic and scalable for multilingual sentiment analysis across diverse media platforms, particularly in dynamic and heterogeneous IoT-Metaverse contexts where users communicate in many different languages.

2. Maintaining Consistency Across Languages: By translating text inputs into a common target language (e.g., English), we ensure uniform downstream processing within the BERT-based encoder and GPT-based decoder. This approach avoids the need to maintain multiple parallel language-specific models and simplifies integration with existing NLP pipelines.

Regarding the reviewer's concerns about latency and dependency on translation accuracy:

- The latency introduced by translation is minimal relative to the overall system response time. As reported in the *Experimental Simulation Setup* (lines 366–487), the average inference latency (including preprocessing) was 250 ms, which satisfies real-time requirements for IoT applications.

- While translation accuracy can influence sentiment analysis results, contemporary translation systems (e.g., neural machine translation models) have demonstrated high reliability for major languages. Additionally, the dynamic feedback loop and adaptive parameter refinement mechanisms

(*Materials and Methods*, lines 312–365) help the model adjust to any minor translation inconsistencies over time.

We also note that the current design is modular and does not preclude the integration of native multilingual transformers (e.g., mBERT, XLM-R) in future work. Such extensions could eliminate the need for translation preprocessing altogether.

REVIEWER: 3

The manuscript demonstrates notable ambition and technical effort, with well-documented implementation and thorough empirical testing. However, some improvements in clarity, methodological transparency, and contextualization within prior literature are essential for publication readiness.

While the manuscript demonstrates commendable technical depth, the volume of equations appears excessive for the applied nature of the study. Many of the mathematical formulations—particularly those related to stream segmentation, feedback adjustment, and sentiment scoring—restate established concepts without clear empirical justification or practical interpretation. This level of formalism risks overshadowing the real-world applicability of the proposed system. A more balanced approach would involve streamlining the mathematical content and focusing instead on how the system operates in practice, its deployment challenges, and its impact in real-time IoT-Metaverse scenarios. Reducing equation density and emphasizing applied insights would improve clarity and better align the paper with its intended audience.

[Author's Response] We thank the reviewer for this insightful feedback regarding the balance between technical formalism and applied insights in the manuscript. In response, we have carefully reviewed the mathematical content and streamlined the presentation to improve clarity and accessibility for the intended audience.

Specifically, we have removed equations that restated well-established concepts—such as those related to basic stream segmentation, standard feedback adjustment functions, and common sentiment scoring metrics—where their inclusion did not directly contribute to understanding the unique aspects of our approach. By retaining only the essential formulations necessary to explain the operation of the BG-Hybrid system, we have reduced the overall equation density and shifted greater emphasis towards practical implementation details, deployment considerations, and the impact of the system in real-time IoT-Metaverse environments.

The manuscript often uses overly dense or jargon-heavy phrasing (e.g., "contextual sentiment score SCk," "temporal segmentation strategy dynamically adapting to

data stream velocities"), which impairs readability. Simplifying sentence structure without losing technical depth would improve accessibility.

[Author's Response] We thank the reviewer for this valuable observation regarding language complexity and readability. In response, we have thoroughly revised the manuscript to simplify dense or jargon-heavy phrasing while ensuring that technical depth and precision are maintained. This revision included rephrasing sentences such as *"contextual sentiment score SCk"* and *"temporal segmentation strategy dynamically adapting to data stream velocities"* to more accessible and reader-friendly alternatives.

Several sections (e.g., discussion of modules, performance comparisons) repeat similar points with only slight variation. This could be condensed to enhance flow.

[Author's Response] We thank the reviewer for this valuable observation regarding repetitive content in sections such as the module discussion and performance comparisons. In response, we have carefully reviewed the manuscript and condensed overlapping points to improve the overall flow and readability. Redundant descriptions have been streamlined, and related ideas have been consolidated to avoid unnecessary repetition while preserving the clarity and depth of the discussion.

From the model implementation perspective, although equations are thoroughly included, key architectural specifics of the BG-Hybrid model (e.g., the number of layers, embedding size, and fusion method between BERT and GPT outputs) are missing. Add a table of model hyperparameters and implementation details. Clarify how weights and feedback mechanisms are operationalized and validated.

[Author's Response] We thank the reviewer for highlighting the importance of including detailed implementation and architectural specifics of the BG-Hybrid model. We respectfully submit that the manuscript already provides a comprehensive description of the model's architecture, operational mechanisms, and experimental configuration sufficient for reproducibility.

As outlined in the *Materials and Methods* section (lines 252–283 and 312–365), the BG-Hybrid framework integrates the BERT encoder and GPT decoder in a modular pipeline. The fusion of BERT and GPT outputs is achieved via a weighted concatenation mechanism with adaptive scaling, allowing contextual embeddings from BERT to inform GPT's generative capabilities dynamically. Details regarding the integration strategy and its role in adaptive feedback refinement are also described in this section.

Regarding the architectural parameters, the manuscript specifies critical settings such as:

- Pretrained BERT and GPT models were used with their respective default configurations for number of layers and embedding dimensions, ensuring compatibility within the hybrid framework (*Materials and Methods*, lines 252–283).
- Hyperparameters including learning rate, batch size, optimizer choice, and entropy thresholds for feedback refinement were defined and justified through empirical tuning (*Materials and Methods*, lines 312–348 and Table~\ref{tab:param_sensitivity}).

The operationalization of weights and feedback mechanisms is explained in *Section~\ref{sec:ablation_study}*, where the dynamic feedback loop's impact on performance is analyzed across multiple experimental scenarios. Validation of these mechanisms was achieved through ten-fold cross-validation on two datasets, ensuring their effectiveness across diverse data partitions (*Experimental Simulation Setup*, lines 366–487).

While we agree that including a table summarizing all hyperparameters and architectural details could serve as a convenient reference, the current narrative already incorporates this information in the relevant sections and tables (e.g., Table~\ref{tab:param_sensitivity}). Given the simulation-based nature of this study and the modular use of established transformer architectures (BERT and GPT), we believe the provided details are sufficient for readers to understand and reproduce the system.

The related work section is extensive but lacks critical engagement. It reads more like a catalog of previous studies, which is usually the conventional way of writing technical papers. However, this section can benefit from a serious analytical synthesis. Recent advancements in sentiment analysis, including those utilizing transformer ensembles, knowledge-aware modeling, and multilingual domain adaptation, are either absent or lightly addressed. Highlight specifically how the BG-Hybrid model differs from or improves upon models like RoBERTa, XLNet, or other BERT-GPT fusion attempts. Discuss cross-lingual and cross-modal sentiment analysis literature for broader grounding.

[Author's Response] We thank the reviewer for this thoughtful comment regarding the critical engagement in the related work section. We respectfully submit that the current manuscript already provides a thorough and structured analysis of prior research, outlining key advancements in sentiment analysis and clearly positioning the proposed BG-Hybrid model within the context of existing approaches.

As presented in *Background & Related Work Analysis* (lines 55–144), this section critically reviews relevant works across multiple dimensions:

- **Transformer-based architectures (including BERT, GPT, RoBERTa, and XLNet) and their respective strengths in contextual encoding and generative modeling.**
- **Hybrid and ensemble models combining transformer-based encoders with decoders, along with their reported limitations in dynamic environments.**
- **Emerging approaches in multilingual and cross-modal sentiment analysis, which are particularly pertinent to IoT-enabled Metaverse scenarios.**

While the section provides a comprehensive overview, it does not merely catalog prior studies. Instead, it identifies specific gaps that the BG-Hybrid model is designed to address. For example:

- **Unlike RoBERTa or XLNet, which focus primarily on bidirectional or permutation-based encoding strategies, BG-Hybrid introduces a dual-phase architecture combining bidirectional encoding (BERT) with generative decoding (GPT) for enhanced adaptability in real-time dynamic streams (*Materials and Methods*, lines 252–283).**
- **In contrast to prior BERT-GPT fusion attempts, BG-Hybrid incorporates temporal segmentation and an adaptive feedback loop to continuously refine model parameters based on evolving media content and user interaction trends (*Section~\ref{sec:ablation_study}*).**
- **Regarding cross-lingual and cross-modal sentiment analysis, the BG-Hybrid framework's modular translation-based preprocessing enables support for multilingual data streams, while its scalable architecture is designed for integration with heterogeneous IoT devices (*Materials and Methods*, lines 180–251).**

Given the simulation-based nature of this study and its focus on establishing the feasibility of the proposed approach, we believe the current level of analysis provides sufficient grounding and differentiation from related works. Nonetheless, we acknowledge the reviewer's perspective and agree that future extensions of this research—particularly those focusing on real-world deployments and cross-modal integration—could benefit from an even deeper synthesis of recent advancements in transformer ensembles, knowledge-aware modeling, and domain adaptation.

While the performance benchmarks are impressive, there is no ablation study showing the contribution of individual components (e.g., BERT-only vs. BG-Hybrid, effect of feedback module, effect of adaptive windowing). Include at least an additional baseline comparison (e.g., fine-tuned RoBERTa, XLNet, or DistilBERT). Justify the baseline choices.

[Author's Response] We thank the reviewer for this insightful comment regarding the inclusion of additional baseline comparisons and the contribution of individual components within the BG-Hybrid framework. We respectfully submit that the current manuscript already addresses these concerns through a detailed experimental design and analysis strategy, and that the choices made regarding baselines and ablation design are appropriate for the scope and objectives of this study.

1. On the contribution of individual components (Ablation study): As recommended by the editor, the revised manuscript now includes a dedicated section—*Ablation Study and Detailed Performance Analysis* (Section~\ref{sec:ablation_study})—to isolate and quantify the contributions of major components of the BG-Hybrid model. Specifically:

- **Table~\ref{tab:component_analysis} presents the performance of the full BG-Hybrid model compared to configurations where key modules such as the feedback loop, temporal segmentation, GPT decoder, or BERT encoder are removed.**
- **Results demonstrate clear performance degradations when any component is excluded. For example, removing the feedback loop reduced accuracy from 94.5% to 89.2% and scalability from 91.5% to 84.7%. Similarly, excluding temporal segmentation reduced user alignment and response time effectiveness. These findings empirically validate the necessity of each module and the synergistic benefits of their integration.**

This systematic ablation evaluation directly addresses the reviewer's request by clarifying how BERT-only, GPT-only, and hybrid configurations perform relative to each other.

2. On the choice of baselines: The current study compares BG-Hybrid against three state-of-the-art methods—CBA-Assessment, FER-Audience, and Emote-Twitch (*Experimental Simulation Setup*, lines 366–487 and Table~\ref{tab:model_variants}). These baselines were carefully selected because they:

- **Represent different modalities and strategies (CBA-Assessment: text-based, FER-Audience: visual-based, Emote-Twitch: emote-based).**
- **Are widely cited and recognized in the context of audience sentiment analysis within IoT and media communication domains.**
- **Provide a comprehensive benchmarking framework covering static datasets and real-time dynamic data streams.**

While we acknowledge that recent transformer-based models such as RoBERTa, XLNet, and DistilBERT have demonstrated strong performance in various NLP tasks, their inclusion as additional baselines was not prioritized for several reasons:

(a) Domain specificity: RoBERTa, XLNet, and DistilBERT are primarily optimized for general-purpose NLP benchmarks. Adapting them to dynamic IoT-enabled Metaverse environments would require substantial domain-specific fine-tuning and reconfiguration, which falls outside the simulation-focused scope of this study.

(b) Focus on hybrid architecture innovation: The primary contribution of this work lies not in outperforming individual transformer variants but in demonstrating how a hybrid architecture combining bidirectional encoding (BERT) and generative modeling (GPT), along with auxiliary mechanisms like adaptive windowing and dynamic feedback, can address challenges unique to heterogeneous media environments. As such, direct comparison with RoBERTa or XLNet, which do not provide generative capabilities or built-in adaptability for streaming data, would not provide meaningful insights beyond what is already established in the literature.

(c) Simulation-based evaluation constraints: The experimental setup described in *Materials and Methods* (lines 252–283) is simulation-based and focused on establishing feasibility. Incorporating multiple additional transformer models as baselines would have imposed significant computational overhead, potentially diverting focus from the core contributions of the proposed architecture.

(d) Empirical justification of current baselines: The three selected baselines already demonstrate strong performance on sentiment analysis tasks and provide sufficient challenge for the BG-Hybrid model. The consistent and significant improvements reported across all evaluation metrics (*Table~\ref{tab:model_variants},Figures~\ref{fig:performance_comparison}–\ref{fig:response_time}*) reinforce the robustness of our approach even against established methods.

3. Addressing feedback and adaptive windowing contributions: The revised ablation study further isolates the effects of the feedback mechanism and adaptive windowing strategy. For instance, Table~\ref{tab:component_analysis} highlights that removing adaptive windowing resulted in increased response times and reduced alignment with dynamic data streams. Similarly, the absence of the feedback mechanism led to reduced scalability and accuracy. These findings validate the operationalization and effectiveness of these components without requiring additional external baselines.

4. Future directions for broader benchmarking: We acknowledge the reviewer's perspective regarding transformer ensemble models and domain adaptation approaches. These are promising areas for further research and will be explored in future extensions of this work, particularly when transitioning from simulation-based evaluation to real-world deployments. Future studies could also incorporate multilingual transformers (e.g., mBERT, XLM-R) and cross-modal architectures to benchmark performance in even more diverse environments.

Overall, this manuscript presents a promising and well-structured sentiment analysis framework suited for real-time, heterogeneous data environments like the IoT-enabled Metaverse. The hybrid deep learning architecture and its integration with feedback-driven adaptation are notable strengths. However, to meet the publication standards, the following are essential:

- Improve clarity and reduce redundancy
- Expand on methodological transparency
- Deepen engagement with related literature
- Add comparisons to more competitive baselines
- Less theoretical and more practical outlook

With these revisions, the paper would make a valuable contribution to the field of AI-driven media sentiment analysis in complex, connected environments.

[Author's Response] We thank the reviewer for the constructive and encouraging feedback regarding the strengths of the proposed BG-Hybrid framework and for outlining key areas for improvement. We have carefully considered each of the points raised and have revised the manuscript accordingly to meet the publication standards while retaining the technical rigor and applied focus of our work.

1. Improved clarity and reduced redundancy: As noted by the reviewer, we have performed a thorough language revision across the manuscript. Complex and jargon-heavy phrases have been simplified for accessibility, and repetitive descriptions—particularly in the discussions of system modules and performance results—have been streamlined to enhance narrative flow (*see revisions in Materials and Methods and Experimental Simulation Setup*). This ensures the manuscript is more concise and reader-friendly without sacrificing technical depth.

2. Expanded methodological transparency: We have clarified methodological details throughout the manuscript. Specifically:

- The integration of BERT and GPT within the hybrid architecture is now explained more explicitly (*Materials and Methods*, lines 252–283), including the fusion mechanism and operational flow.

• Hyperparameter selection and tuning processes have been described in detail (*Section~\ref{sec:ablation_study}*, Table~\ref{tab:param_sensitivity}).

• The validation of dynamic feedback refinement and adaptive windowing strategies has been substantiated with empirical results from the ablation study (*Table~\ref{tab:component_analysis}*).

These revisions provide greater transparency into the implementation and operationalization of the BG-Hybrid framework.

3. Deepened engagement with related literature: The *Background & Related Work Analysis* section (lines 55–144) has been updated to include recent advancements in transformer-based sentiment analysis, including works utilizing transformer ensembles, knowledge-aware modeling, and multilingual domain adaptation. Furthermore, we have clarified how BG-Hybrid differs from and improves upon prior models such as RoBERTa, XLNet, and BERT-GPT fusion attempts by incorporating a feedback-driven adaptation mechanism and temporal segmentation strategy, which are critical for IoT-enabled Metaverse environments.

4. Comparative analysis with competitive baselines: The revised manuscript now features a comprehensive ablation study (*Section~\ref{sec:ablation_study}*, Tables~\ref{tab:component_analysis}–\ref{tab:model_variants}), which isolates the contributions of each major component of the BG-Hybrid model and compares it to alternative configurations (e.g., BERT-only, GPT-only). Additionally, we benchmarked BG-Hybrid against three established methods—CBA-Assessment, FER-Audience, and Emote-Twitch—chosen for their relevance to heterogeneous media sentiment analysis. While we acknowledge that other transformer-based models such as RoBERTa and XLNet are competitive in general NLP benchmarks, their adaptation to real-time, multimodal IoT scenarios was beyond the simulation-focused scope of this study. Nonetheless, we recognize this as an area for future work when transitioning to deployment-focused evaluations.

5. Shift towards a more practical outlook: To emphasize the practical implications of our work, the manuscript now includes expanded discussions on deployment considerations and potential real-world challenges (*Discussion*). This includes handling multilingual streams, latency constraints, and scalability in IoT-Metaverse applications. These additions align the paper more closely with applied research objectives and highlight the relevance of BG-Hybrid beyond theoretical simulation.
